# Lethal Effect of Total Dissolved Gas-Supersaturated Water with Suspended Sediment on River Sturgeon (*Acipenser dabryanus*)

**DOI:** 10.1038/s41598-019-49800-y

**Published:** 2019-09-16

**Authors:** Xiaoqing Liu, Na Li, Cuixia Feng, Chenghua Fu, Quan Gong, Jiansheng Lai, Zhu Jiang, Yao Yang, Haoran Shi

**Affiliations:** 1 Key Laboratory of Fluid and Power Machinery, Ministry of Education, School of Energy and Power Engineering, Xihua University, Chengdu, 610039 China; 2 Fisheries Institute, Sichuan Academy of Agricultural Sciences, Chengdu, 611731 China

**Keywords:** Ichthyology, Freshwater ecology

## Abstract

High total dissolved gas (TDG) levels and excessive suspended sediment (SS) concentrations pose serious threats to fish survival during flood season. However, little information is available on the effects of TDG supersaturation with varying SS concentrations on fish. In this study, laboratory experiments were performed to investigate the effects of TDG supersaturation with varying SS concentrations on five-month-old river sturgeons (*Acipenser dabryanus*). The test fish were exposed to combinations of SS concentrations (0, 200, 600 and 1,000 mg/L) and TDG levels (125, 130, 135 and 140%), and their mortality and median lethal time (LT_50_) were quantified. The fish showed abnormal behaviors (e.g., quick breathing, fast swimming and an agitated escape response) and symptoms of gas bubble disease (GBD). SS increased the mortality of river sturgeon exposed to TDG supersaturation. Furthermore, the LT_50_ values at 125% TDG were 4.47, 3.11, 3.07 and 2.68 h for the different SS concentrations (0, 200, 600 and 1,000 mg/L, respectively), representing a significant decrease in LT_50_ with increasing SS. However, at higher TDG levels (130–140%), there was no significant increase in LT_50_ with increasing SS. Therefore, river sturgeon showed weak tolerance of TDG-supersaturated water with SS.

## Introduction

In the last ten years, seven high dams (higher than 250 m), such as the Xiaowan Dam, Xiluodu Dam, and Jinping Dam, have been built in China. When these high dams discharge flood water, total dissolved gas (TDG) supersaturation commonly occurs. A high level (128%) has been reported 180 km downstream of the Three Gorges Dam^1^. In addition, the discharged flood contains a large quantity of sediment. The cumulative effects of high TDG levels and suspended sediment (SS) might seriously influence aquatic ecosystems^2^. Previous studies indicated that TDG supersaturation threatened the survival of fish dwelling downstream of dams due to gas bubble disease (GBD)^3,4^. Similarly, mortality due to GBD was found in the Columbia River Basin^5–8^. Recently, many studies on the effect of TDG supersaturation on endemic species, such as rock carp (*Procypris rabaudi* Tchang), Chinese sucker (*Myxocyprinus asiaticus*) and David’s schizothoracin (*Schizothorax davidi*), in the Yangtze River have been conducted^9–12^. In 2014, China Central Television (CCTV) reported that a large number of fish died from TDG supersaturation in the Jinsha River during flood discharge^13^. Furthermore, previous studies showed that SS at high concentrations had adverse effects on arctic grayling (*Thymallus arcticus*), Coho salmon (*Oncorhynchus kisutch)* and coral reef damselfish (*Acanthochromis polyacanthus*)^14–16^. Increasing SS reduced the foraging and growth of coral reef damselfish (*Acanthochromis polyacanthus*)^16^. The survival of common smelt (*Retropinna retropinna*) and Coho salmon (*Oncorhynchus kisutch)* was reduced by increasing SS concentrations^17,18^.

The mean sediment content in the flood season remained high in the Yangtze River from 1956 to 2010 (e.g., 535 mg/L in the Min River among the tributaries of the Yangtze River and 1,660 mg/L in the Jinsha River in the upper Yangtze River). Moreover, TDG levels are mostly between 120% and 140% during flood discharge^19,20^. However, the potential harm due to higher SS concentrations in addition to TDG supersaturation has not been previously considered. The population size of the river sturgeon, a class national protected animal in China, has decreased greatly, and the species is facing extinction owing to habitat destruction. Therefore, the aim of this study was to investigate the effect of TDG supersaturation with varying SS concentrations on river sturgeon from the upper Yangtze River. The data resulting from this study can provide a scientific basis for the protection of river sturgeon and other species and will play an important role in establishing appropriate environmental standards to improve fish habitat.

## Materials and Methods

### Ethics statement

The experimental proposal was approved by the Ethics Committee for Animal Experiments of the Sichuan Academy of Agricultural Sciences and Xihua University. All experiments were executed in accordance with the animal management regulations of Sichuan Province in China.

### Test fish and experimental sediment

The river sturgeon is a rare species in the upper Yangtze River and inhabits 8–10 m shallow waters. The species usually moves in slow-flowing water and can tolerate the wide water temperatures (1–32 °C). River sturgeon grows fast and the water temperatures of 18–25 °C increase the growth. However, the growth rate of river sturgeon slows down when the water temperature is below 13 °C or above 28 °C. The species is bred at the Sichuan Fisheries Research Institute under government authorization. In this experiment, five-month-old juveniles were acquired from the Sichuan Fisheries Research Institute. Healthy juveniles (mean fork length ± standard deviation (SD), 13.4 ± 0.7 cm; mean weight ± SD, 7.1 ± 1.2 g) with a uniform body size were selected. Experimental sediments were collected from the Jinsha River. Based on data from China’s river sediment communique^21^, the annual average sediment diameter was approximately 7 μm in the Yangtze River in 2000–2010. Sediment with a median diameter of 7 μm was chosen for the experiment, matching the particle size in the Yangtze River.

### Experimental device

The experimental device was mainly composed of a water flume, a pressure vessel, a pump, an air compressor and experimental tanks^22^. The sediment was maintained in suspension by using a submerged pump placed in the bottom of the water flume. Turbid water from the water flume was sent into the pressure vessel and mixed with air from the compressor to create high TDG-supersaturated water with SS. The resulting water was mixed with sandy water with 100% TDG to create different levels of TDG-supersaturated water with SS. The water temperature was maintained at 21–22 °C by the temperature controller. A Point Four Tracker (Point Four Systems Inc., Canada) was used to measure the TDG level and water temperature. The pH and dissolved oxygen (DO) were monitored by using a digital pH meter (JENCO Model 6010, China) and a dissolved oxygen meter (Oxi 3210 SET 3 Inc., Germany), respectively. Sediment concentration and turbidity were measured with a turbidimeter (Hach 2100, China).

### Acclimation conditions

The test fish were acclimated for five days in a tank (720 L) in the Key Laboratory of Fluid and Power Machinery, Ministry of Education (Xihua University, China). During the acclimation period, the flow-through water temperature was 21–22 °C, the pH value was 7.1–7.4, and the DO was 7.2–8.0 mg/L. Fish were given *Limnodrilus hoffmeisteri* as food every morning and afternoon. The rearing tank was cleaned at the same time every day.

### Acute lethal experiment

Based on field observational data, the TDG levels are mostly below 140% during flood discharge^19,20^. In this study, we set the TDG levels at 125, 130, 135 and 140% and investigated the influence of TDG exposure with SS concentrations of 0, 200, 600 and 1,000 mg/L. Control groups were held at a 0 mg SS concentration with the four TDG levels. Two parallel experiments were performed for each experimental group. Following acclimation and preparation of the experiment, 60 fish were moved into each experimental tank (length: 1 m; width: 0.6 m; water depth: 0.5 m). During the trial, the water temperature, pH and DO were kept consistent with those of the acclimation period. The flow velocity of circulating water was approximately 0.5 cm/s in each experimental tank. In addition, abnormal behaviors and symptoms of GBD of the test fish were continuously observed. The time of death of each fish was also recorded during the trial. Mortality and median lethal time (LT_50_; the exposure time at which the mortality of fish reached 50% at a specific TDG level) were determined to investigate the effect of TDG-supersaturated water with SS on river sturgeon.

### Statistical analysis

The LT_50_ was calculated by the method of Shetty and Alwar^23^. The difference in LT_50_ among TDG levels was analyzed using one-way analysis of variance (ANOVA) followed by a least significant difference (LSD) test. Dunnett’s T_3_ test was used when the assumption of homogeneity of variance was not met. Regression analysis was used to investigate the relationship between LT_50_ and SS at different TDG levels. Furthermore, a two-way ANOVA followed by a post hoc multiple comparison test (Tukey test) was executed in SPSS to determine the effect of TDG supersaturation with varying SS concentrations on river sturgeon. Data were expressed as the mean ± SD, and the significance level was set at P < 0.05.

## Results

### Abnormal behaviors and signs of GBD

**Figure 1:**
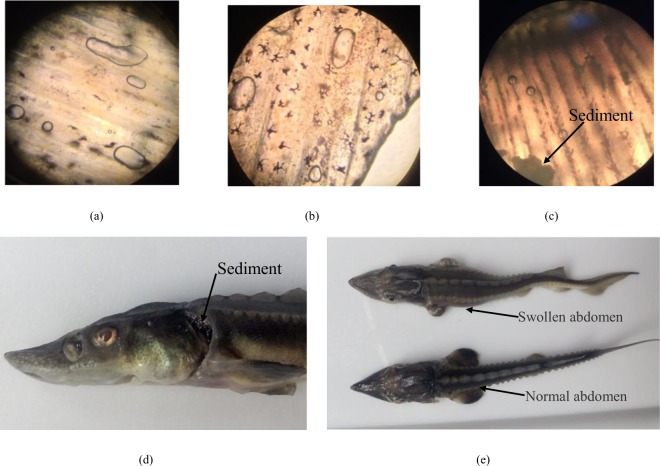
GBD symptoms of river sturgeon observed in the lethal experiment. (**a**) Bubbles on pectoral fins. (**b**) Bubbles on dorsal fins. (**c**) Bubbles and sediment in gills. (**d**) Sediment in gills. (**e**) Swollen abdomen and normal abdomen.

The fish first exhibited abnormal behaviors, such as quick breathing, fast swimming and an agitated escape response, after 20 minutes in the 140% TDG treatment. In addition, many bubbles were found on the fish skin. Over the next 15 minutes, some of the fish lost their equilibrium, and other fish put their heads out of the water from time to time. After an hour in the 140% TDG treatment, the fish became moribund and died. Dead fish either sank to the tank floor or floated. The above abnormal behaviors were also found at other TDG levels (125, 130 and 135%). Furthermore, microbubbles were observed in the gills and on the pectoral fins and dorsal fins with a microscope (SZM-45T2, China) (Fig. 1a–c). Sediments were apparent in the gills (Fig. 1c,d). Most sturgeons (93%) exposed to TDG-supersaturated water exhibited a swollen abdomen (Fig. 1e).

### Mortality at different TDG levels and SS concentrations

**Figure 2:**
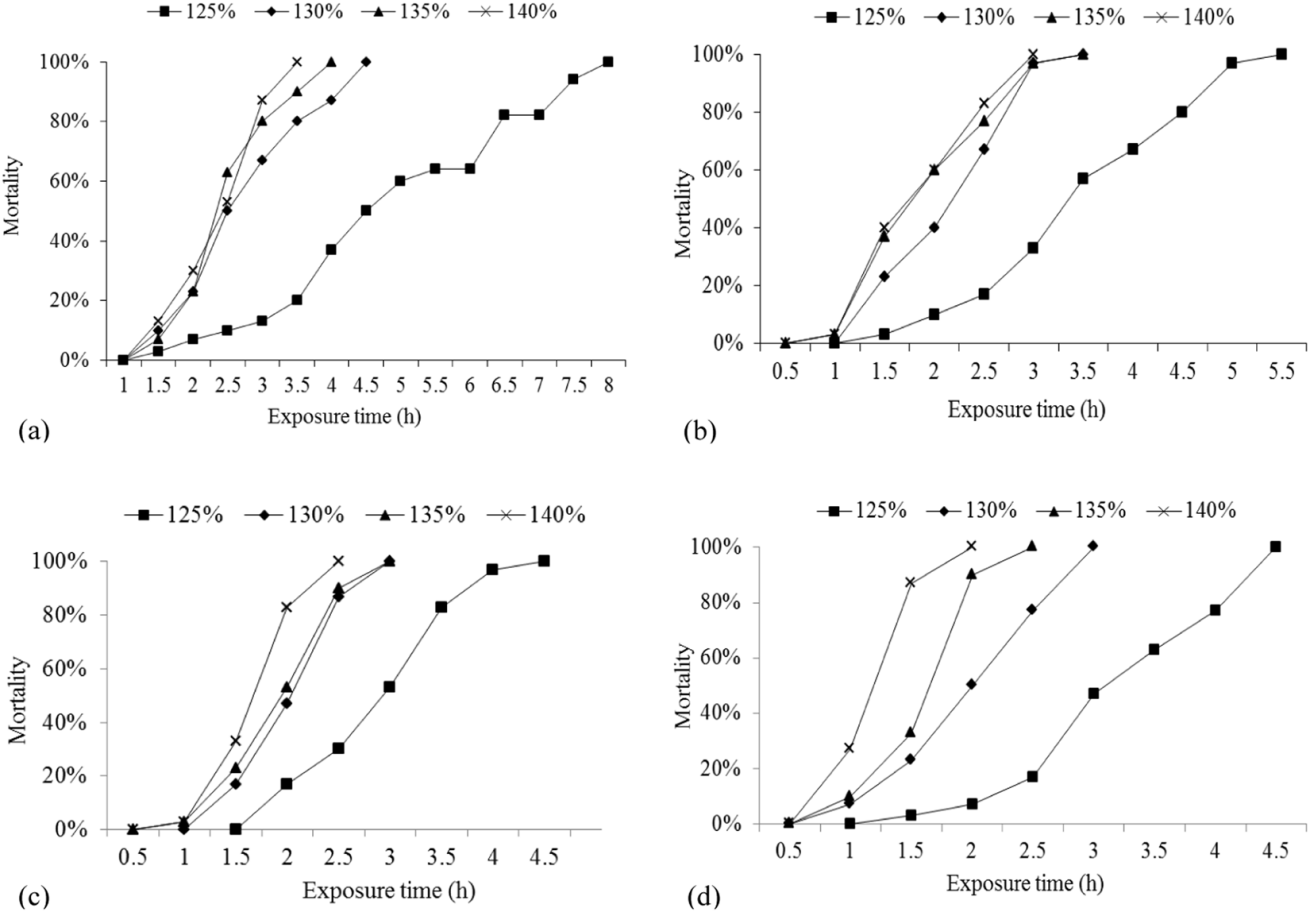
Mortality of river sturgeon exposed to TDG levels of 125–140% at each sediment concentration: (**a**) 0 mg/L; (**b**) 200 mg/L; (**c**) 600 mg/L; (**d**) 1,000 mg/L.

The relationships among the mortality of river sturgeon, TDG levels and exposure times with different sediment concentrations are shown in Fig. 2. Mortality reached 100% in 7.7 h when the river sturgeons were exposed to water with a TDG level of 125% (Fig. 2a). By comparison, survival time (at 0% survival) was shorter with higher TDG at 130–140%. In turbid water (200 mg/L), the survival times of river sturgeon in groups with 125, 130, 135 and 140% TDG levels were 5.3, 3.2, 3.1 and 2.6 h, respectively (Fig. 2b). Survival time (at 0% survival) fell from 4.3 to 2.2 h at 600 mg/L (125~140% TDG) (Fig. 2c) and to 1.7 h at 1,000 mg/L (SS) and 140% TDG (Fig. 2d). Increased sediment concentrations resulted in increased mortality at the same TDG levels. Similarly, increased TDG levels reduced survival times at the same sediment concentrations.

### Median lethal time (LT_50_)

**Figure 3:**
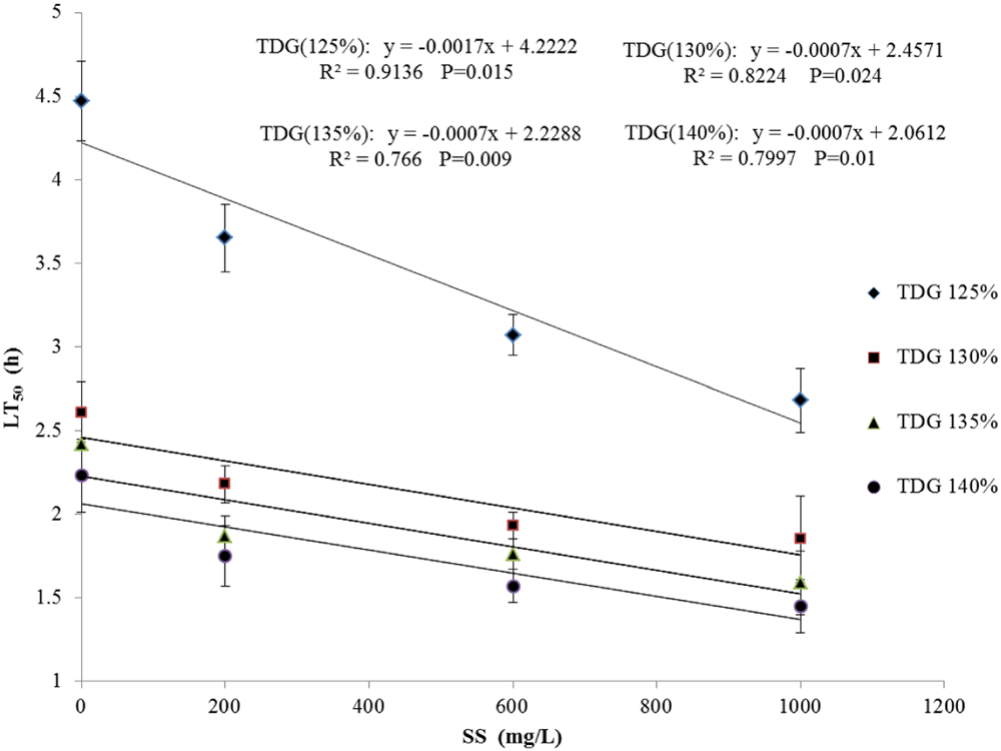
Linear correlation between the LT_50_ and SS concentrations at different TDG levels. Regression analysis was used to investigate the relationship. In these equations, *y* is the LT_50_ value, *x* is SS concentration, and *R*^2^ is the regression coefficient. P < 0.05 presents a significant negative linear correlation between the LT_50_ and SS concentrations at different TDG levels. The data are presented as the mean ± SD (*n* = 2).

The LT_50_ of the river sturgeons exposed to TDG-supersaturated water with varied SS concentrations is shown in Table 1. Based on two-way ANOVA, SS concentration and TDG level had significant effects on the LT_50_ of river sturgeon (SS: *df* = 3, F = 25.79, *P* = 0.0001 < 0.05, partial eta squared = 0.829; TDG: *df* = 3, F = 77.08, *P* = 0.0001 < 0.05, partial eta squared = 0.935). The effect of TDG on the LT_50_ of the river sturgeons was greater than that of SS. However, the interaction between these two variables was not significant (*df* = 9, F = 1.542, *P* = 0.215 > 0.05, partial eta squared = 0.465). In addition, at 125% TDG, the LT_50_ declined significantly with increasing SS concentration (F_(3,4)_ = 16.35, *P* = 0.01 < 0.05). The LT_50_ values decreased by 40% at 0 mg/L (4.47 h) compared with those at 1,000 mg/L (2.68 h). In the other TDG groups, no difference in LT_50_ was observed (130% TDG: F_(3,4)_ = 3.946, *P* = 0.109 > 0.05; 135% TDG: F_(3,4)_ = 4.072, *P* = 0.104 > 0.05; 140% TDG: F_(3,4)_ = 4.04, *P* = 0.105 > 0.05). The lowest LT_50_ value (1.45 h) was reached at 1,000 mg/L SS and 140% TDG. As shown in Fig. 3, the LT_50_ values exhibited a significant negative linear correlation with the SS concentrations at different TDG levels (125% TDG: $y=-\,0.0017x+4.2222$, *R*^2^ = 0.9136, *P* = 0.015; 130% TDG: $y=-\,0.0007x+2.4571$, *R*^2^ = 0.8224, *P* = 0.024; 135% TDG:$y=-\,0.0007x+2.2288$, *R*^2^ = 0.766, *P* = 0.009; and 140% TDG: $y=-\,0.0007x+2.0612$, *R*^2^ = 0.7997, *P* = 0.01, where *y* is the LT_50_ value, *x* is SS concentration, and *R*^2^ is the regression coefficient).

**Table 1:** The LT_50_ of river sturgeon at different SS concentrations and TDG levels, presented as the average (±SD, *n* = 2) of the two parallel experiments within each combination of TDG and SS. There were 60 fish in each treatment combination.

SS (mg/L)	TDG levels			
	125%	130%	135%	140%
0	4.47 ± 0.24	2.61 ± 0.18	2.42 ± 0.19	2.23 ± 0.22
200	3.65 ± 0.20	2.18 ± 0.11	1.87 ± 0.12	1.75 ± 0.18
600	3.07 ± 0.12	1.93 ± 0.08	1.76 ± 0.20	1.57 ± 0.10
1,000	2.68 ± 0.19	1.85 ± 0.26	1.59 ± 0.19	1.45 ± 0.16

The interaction between these two variables was not significant in LT_50_ based on two-way ANOVA.

## Discussion

Previous studies demonstrated that GBD caused by TDG supersaturation resulted in the mortality of fish and threatened their survival downstream of dams during flood discharge^24–26^. Mortality increased with TDG level in rock carp (*Procypris rabaudi* Tchang), Chinese sucker (*Myxocyprinus asiaticus* Bleeker) and Prenant’s schizothoracin (*Schizothorax prenanti*)^9,11,22,27^. Some typical symptoms of GBD, such as exophthalmos, shedding of scales, swelling of the swim bladder, and bubbles in the gills and fins, have been reported^7,9,11,28^. Hemorrhages in the gills and fin of Chinese species (Prenant’s schizothoracin (*Schizothorax prenanti*) and silver carp (*Hypophthalmichthys molitrix*)) occurred because TDG in the blood decreased blood circulation frequency and led to blood stasis^22,27,29^. There were some similar signs of GBD in the present study. TDG supersaturation resulted in a swollen abdomen in the river sturgeon due to GBD. Moreover, the present study also detected the abnormal behaviors of breathing quickly, swimming faster and exhibiting an agitated escape response, which were also observed in past studies^11,27^.

Different species have different tolerances of TDG supersaturation. In the study of Beeman *et al*.^26^, the LT_50_ values of five native fish in the Columbia River (largescale sucker, *Catostomus macrocheilus*; longnose sucker, *Catostomus catostomus*; northern pikeminnow, *Ptychocheilus oregonensis*; redside shiner, *Richardsonius balteatus*; and walleye, *Sander vitreus*) were above 15 and 9.5 h at TDG levels of 125 and 130%, respectively. Upstream in the Yangtze River, the LT_50_ values of rock carp were 15.4, 8.2, 6.6 and 3.5 h at 125, 130, 135 and 140% TDG levels, respectively^9^. At the same TDG levels, the LT_50_ of Prenant’s schizothoracin was reduced by 52% from 9.66 to 4.61 h^27^. The LT_50_ values of Chinese suckers were 43.4, 9.2, 7.8 and 4.7 h, respectively^11^. In our study, the LT_50_ values of the river sturgeon were 4.47, 2.61, 2.42 and 2.23 h at the same TDG level range (125, 130, 135 and 140%, respectively). Compared with the above fish, the river sturgeon showed considerably lower tolerance of TDG-supersaturated water. Thus, it would be injured easily by TDG supersaturation.

High SS concentrations have a negative effect on the foraging, growth, and mortality of juvenile fish^14,15,17,18,27^. However, the effect of TDG supersaturation on fish at varying SS concentrations has been little studied. Zhang *et al*. (2014) investigated the interactive effect of TDG and SS on juvenile Prenant’s schizothoracin and showed that increased SS decreased the survival time of juveniles exposed to TDG-saturated water^29^. In the present study, an increase in the SS level also resulted in an increase in the mortality of river sturgeon exposed to TDG-saturated water (Fig. 2), in agreement with the results reported by Zhang *et al*. (2014)^29^. We speculate that a high SS concentration might block fish gills, impede the exchange of oxygen and accelerate death^30^. Furthermore, the LT_50_ of juvenile Prenant’s schizothoracin was 6.55 h at the highest TDG level (140%) and the highest SS concentration (80 mg/L)^29^. Our results showed that the LT_50_ of river sturgeon was 1.45 h at 140% TDG and 1,000 mg/L SS. This difference between Prenant’s schizothoracin and river sturgeon might be related to SS concentration and their tolerances of TDG-supersaturated water with SS. As shown in Table 1, an increase in SS concentration from 0 to 1,000 mg/L strongly reduced the LT_50_ at 125% TDG. The SS and TDG levels were of equal importance for the survival of river sturgeon exposed to TDG-supersaturated water with SS. Furthermore, a relatively high level of TDG (130–140%) induced a low LT_50_ at varying SS concentrations. Thus, TDG level is a main lethal factor for the species. A possible explanation is that emboli were rapidly generated in the fish after exposure to high TDG levels and resulted in death within a short period of time. Thus, TDG-supersaturated water with SS might pose a greater threat than such water without SS to the survival of fish during flood discharge. Such a possibility should not be ignored when planning the management of fish (particularly rare species) dwelling downstream of high dams.

### Conclusion and suggestion

Recently, the effects of TDG supersaturation on fish have received increased attention with the development of more high dams on the Yangtze River. However, few effective measures have been taken to protect fish from harm by TDG supersaturation in China. Our study showed that the influence of TDG-supersaturated water with SS on river sturgeon was greater than that of TDG-supersaturated water alone. This effect should be seriously considered during flood discharges. Therefore, the results of this study can offer important information for the protection of fish and the establishment of effective measures for reservoir regulation in the flood season. However, our study was carried out in a laboratory, and the results cannot be directly applied to the protection of wild species in the Yangtze River. In future studies, the tolerance and behaviors of river sturgeon (or other species) should be examined in natural rivers to evaluate the combined effects of TDG supersaturation and SS.
